# Short Alimentary Canal

**Published:** 1914-06

**Authors:** 


**Short Alimentary Canal.** I. O. Palefski of N. Y., N. Y. Med. Jour., April 18, 1914, reports a case in which the end of a 12-foot duodenal tube was passed through the anus after 48 hours. It could be drawn back and forth between the mouth and the anus, easily, only four feet remaining inside the body. Injecting the tube with bismuth emulsion, it could be seen by the X-rays, the large intestine showing the typic square with open base, a few undulations marking the small intestine.

